# T cells suppress memory-dependent rapid mucous cell metaplasia in mouse airways

**DOI:** 10.1186/s12931-016-0446-0

**Published:** 2016-10-20

**Authors:** Hitendra S. Chand, Yohannes A. Mebratu, Marena Montera, Yohannes Tesfaigzi

**Affiliations:** 1 COPD Program, Lovelace Respiratory Research Institute, Albuquerque, NM 87108 USA; 2 Present Address: Department of Immunology, Herbert Wertheim College of Medicine, Florida International University, 11200 SW 8th St, Miami, FL 33199 USA

**Keywords:** Mucous cell metaplasia, Epithelial memory, LPS, T cells, Toll-like receptor 4, Epidermal growth factor receptor

## Abstract

**Background:**

Airway epithelial cells (AECs) are crucial for mucosal and adaptive immunity but whether these cells respond in a memory-dependent manner is poorly studied. Previously, we have reported that LPS intratracheal instillation in rodents causes extensive neutrophilic inflammation and airway epithelial cell hyperplasia accompanied by mucous cell metaplasia (MCM). And the resolution process required a period of 40 d for the inflammation to subside and the lung epithelia to resemble the non-exposed condition. Therefore, the present study investigated the memory-dependent response of airway epithelial cells to a secondary LPS challenge after the initial inflammation was resolved.

**Methods:**

Airway epithelial and mucous cells were assessed in response to a secondary LPS challenge in F344/N rats, and in C57BL/6 wild-type (Foxn1^WT^) and T cell-deficient athymic (Foxn1^nu^) mice that were instilled with LPS or saline 40 d earlier. Epithelial expression of TLR4, EGFR, and phosphorylated-ERK1/2 (pERK) were also analyzed.

**Results:**

LPS-pretreated F344/N rats responded with elevated numbers of AECs after saline challenge and with 3-4-fold increased MCM following the LPS challenge in LPS- compared with saline-pretreated rats. LPS-pretreated rats showed 5-fold higher number of AECs expressing TLR4 apically than saline-pretreated rats. Also, the expression of EGFR was increased in LPS-pretreated rats along with the number of AECs with active or nuclear pERK, and the levels were further increased upon LPS challenge. LPS-pretreated Foxn1^nu^ compared with Foxn1^WT^ mice showed increased MCM and elevated levels of TLR4, EGFR, and nuclear pERK at 40 d after LPS instillation. LPS challenge further augmented MCM rapidly in Foxn1^nu^ compared with Foxn1^WT^ mice.

**Conclusion:**

Together, these data suggest that AECs preserve an ‘innate memory’ that drives a rapid mucous phenotype via spatiotemporal regulation of TLR4 and EGFR. Further, T cells may suppress the sustained elevated expression of TLR4 and EGFR and thereby the hyperactive epithelial response.

**Electronic supplementary material:**

The online version of this article (doi:10.1186/s12931-016-0446-0) contains supplementary material, which is available to authorized users.

## Background

Airway epithelial cells (AECs) preserve a near-sterile microenvironment via mucociliary clearance mechanisms and more importantly, by an adaptive mucosal immune response [1–4]. The conducting airway epithelium consists of basal, ciliated, club (or Clara) and mucous (or goblet) cells [4–6]. In smaller airways, primarily club cells differentiate into mucous cells as an innate immune response to airway injury also referred to as mucous cell metaplasia (MCM) [4, 5, 7–9]; however other epithelial cells can also differentiate into mucous cells [6, 10] . While mucous cell differentiation is vital to pulmonary health, dysregulation can lead to aberrant mucin secretion and obstruction of airways as observed in chronic respiratory diseases [4, 11]. The most abundant mucins secreted by airway epithelial cells (AECs) are MUC5AC and MUC5B, which in combination with other proteins, lipids and glycosylated factors form a mucous layer [12–14]. The airway surface mucous layer not only serves as a barrier but also traps inhaled particles for mucociliary clearance [4, 11, 14]. Several inflammatory factors and toxicants activate the EGFR/ERK pathway and induce MUC5AC and MUC5B expression to alter innate immune responses [4, 11, 13, 15, 16].

The ambient air is contaminated with various organic or inorganic compounds including lipopolysaccharide (LPS), a component of gram-negative bacteria. Our previous studies showed that intratracheal instillation of 1000 μg LPS in rats causes extensive neutrophilic inflammation and epithelial cell hyperplasia accompanied by MCM [7, 17]. The resolution process for this extensive inflammation required a period of 40 d for the inflammation to subside and the lung epithelia to resemble the non-exposed condition [17].

The airway epithelium has evolved several regulatory mechanisms to control hyperreactive inflammatory responses to minimize deleterious effects. Lymphocytes, monocytes, macrophages, and dendritic cells possess ‘dynamic cellular programing’ or ‘memory’ to adjust the immune response to secondary challenges [18–22]. Various animal studies have highlighted the important role of adaptive T cells, both CD4 and CD8 T lymphocytes, and macrophages in regulating immune responses to previously encountered insults [23–26]. However, only few reports have reported on epithelial cell ‘innate-programming’ or ‘memory’ responses [27, 28]. Therefore, the present study was designed to investigate the response of airway epithelium to a secondary LPS challenge. Possible interaction of T cells and AECs in regulating memory responses was investigated by using T cell-sufficient and -deficient mice.

## Methods

### Laboratory animals

All animal studies were carried out using protocols and the facilities pre-approved by the Institutional Animal Care and Use Committee (IACUC) under the approved protocol number FY06-021. Specific pathogen-free F344/NCrR male rats of 6–8 weeks of age were obtained from NCI (Frederick, MD) and C57BL/6 J wild-type (Foxn1^WT^) and the T-cell deficient congenic athymic nude (Foxn1^nu^) mice were purchased from the Jackson Laboratory.

### LPS instillation

**Fig. 1:**
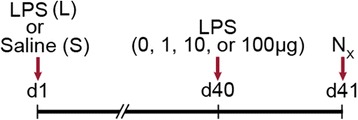
Experimental design for testing the effect of LPS challenge on LPS-pretreated rats. F344/N rats were challenged intratracheally with 1000 μg LPS (L) or saline (S) on d1 and rechallenged with 0, 1, 10 or 100 μg LPS on d40, and the necropsy (N_x_) was performed on d41

F344/N rats were instilled intratracheally with 1000 μg and Foxn1^nu^ and Foxn1^WT^ C57BL/6 J mice were instilled intranasally with 100 μg LPS (*Pseudomonas aeruginosa* serotype 10, lot 31 K4122, 3,000,000 LPS units (EU)/mg, Sigma, St. Louis, MO) or with saline as described previously [17, 29]. Forty days later, rats were instilled with saline (0) or 1, 10, or 100 μg LPS (Fig. 1) when all of the inflammatory responses are resolved [17]. The LPS-pretreated (L) rats were designated as L/0, L/1, L/10, and L/100; and the saline-pretreated (S) rats designated as S/0, S/1, S/10, and S/100. Mice were challenged with saline (0) or 100 μg LPS and were designated as S/0, and S/100 or L/0, and L/100, respectively. Animals were sacrificed 24 h post-instillation, and lung tissues were processed and analyzed.

### Histochemical staining and analysis

Histochemical staining with Alcian Blue (Richard-Allan Scientific) and hematoxylin and eosin (AB-H&E) was carried out as described previously [30]. Airway epithelial cell and mucous cell numbers per mm basal lamina (BL) were measured using the VisioMorph system (Visiopham A/S, Horsholm, Denmark). In all cases, quantification and morphometry was carried out by a person unaware of slide identity. For all of the epithelial cell responses in-vivo we analyzed the large-diameter generation 5 conducting airway along the main axial pathway as described before [7]. Generation 5 was chosen for all of the analyses because those airway regions are predominantly comprise of secretory and ciliated cells. Furthermore, the instilled LPS readily reaches the cells at generation 5, which allows for consistent results. Unless otherwise mentioned, 5-6 animals were used per group.

### Immunofluorescent staining

Lung sections were processed and immunostained as described previously [31]. Sections were probed with anti-Scgb1a1 (Santa Cruz Biotech, CA), anti-Muc5ac (Millipore, CA), and anti-phospho ERK1/2 (Cell Signaling, CA) and the immunopositive cell numbers as well as the mean fluorescence intensity (MFI) were quantified using the NIH ImageJ software.

### Statistical analysis

Grouped results were expressed as means ± SEM and were analyzed using either one-way or two-way analysis of variance. The data were compared with that of S/0 or L/0 groups unless otherwise indicated. In the event that significant main effects were detected (*P* < 0.05), Fisher's least significant difference test was used to differentiate between groups.

## Results

### Rapid and enhanced mucous cell metaplasia following LPS challenge

**Fig. 2:**
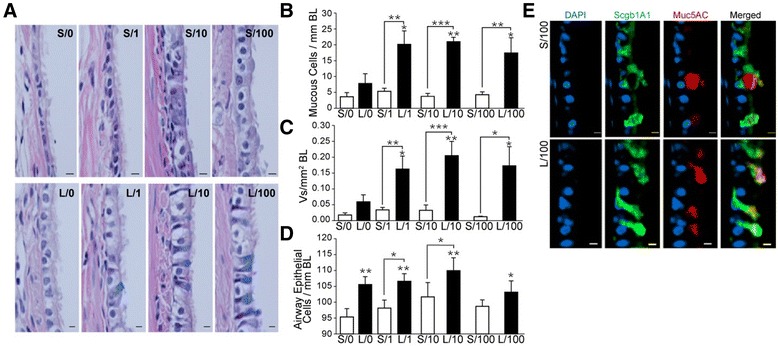
LPS-induced mucous cell metaplasia and mucous production is augmented in LPS-pretreated F344/N rats. **a** Representative photomicrographs of axial airway sections from rats instilled with 0, 1, 10 or 100 μg LPS as secondary challenge following the saline (S/0, S/1, S/10 or S/100) or LPS (L/0, L/1, L/10 or L/100) pretreatment, and stained with Alcian Blue (AB) for acidic mucins and counterstained with hematoxylin and eosin (AB/H&E). **b** Quantification of the number of AB+ mucous cells analyzed per mm basal lamina (BL). **c** Densitometric quantification of the volume of intraepithelial stored mucosubstances (Vs) analyzed per mm^2^ of BL. **d** Quantification of the number of total airway epithelial cells per mm BL. **e** Representative micrographs of axial airway sections from S/100 and L/100 rats. The sections show Scgb1A1 (*green*) and Muc5AC (*red*) positivity with DAPI-stained (*blue*) nuclei. Error bar indicates mean ± SEM (*n* = 5–6 per group). * *P* < 0.05, ** *P* < 0.01, *** *P* < 0.001 compared to S/0 or L/0 group unless otherwise indicated

Although mucous cell (MC) numbers per mm basal lamina (BL) did not reach statistical significance, L/0 compared to S/0 rats displayed increased MCs per mm BL (Fig. 2a and b), and this difference was magnified in L/1, L/10, and L/100 rats compared to their respective S/1, S/10, and S/100 controls (Fig. 2b). The stored mucosubstances quantified as mucus density (V_s_) per mm^2^ BL was also augmented in LPS- compared to saline-pretreated rats by 8–10-fold (Fig. 2c). The total epithelial cells per mm BL was significantly increased in L/0 compared to S/0 rats, and no further increase was observed in L/1, L/10, and L/100 rats (Fig. 2d).

The number of Scgb1A1-positive club cells between saline- or LPS-pretreated rats remained unchanged; however, the number of Muc5AC-positive mucous cells in L/100 compared with S/100 controls were significantly increased (Fig. 2e) while Scgb1A1 expression was markedly reduced upon LPS challenge. Thus, LPS pretreatment in rats caused a rapid increase in mucus-secretory cells.

### Elevated apical expression of TLR4 and EGFR and nuclear phosphorylated-ERK1/2

**Fig. 3:**
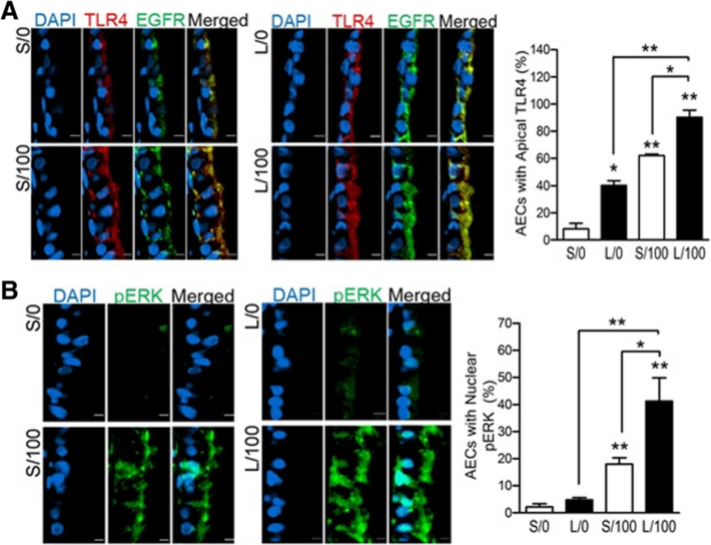
Elevated expression of TLR4, EGFR and phosphorylated-ERK1/2 (pERK) in LPS-pretreated F344/N rats following the second LPS challenge. **a** Quantification of the AECs with apically localized TLR4 and EGFR in S/0, S/100, L/0 and L/100 rats. Representative micrographs of axial airways from S/0, S/100, L0 and L/100 rats with airways stained with DAPI (*blue*) nuclei, TLR4 (*red*), EGFR (*green*) and a merged image. **b** Quantification of the AECs with nuclear phosphorylated-ERK1/2 (p-ERK) in S/0, S/100, L/0 and L/100 rats. Representative micrographs of axial airways stained for pERK (*green*) and DAPI-stained (*blue*) nuclei are shown. Error bar indicates mean ± SEM (*n* = 5–6 per group). * *P* < 0.05, ** *P* < 0.01 compared to S/0 unless otherwise indicated

TLR4 mediates LPS-induced responses [32–34], and the integrated signal transduction via EGFR drives mucous gene expression and MCM [35, 36]. Therefore we analyzed TLR4 and EGFR expression in the AECs of S/0, L/0, S/100, and L/100 rats. EGFR was detected on the basal and intraepithelial junctions of AECs in S/0 rats and slightly more abundant in the apical region of L/0 rats; however, the number of AECs expressing TLR4 apically was 5-fold higher in L/0 than S/0 rats (Fig. 3a, upper panels). The LPS challenge increased the number of cells with apical TLR4 expression in both S/100 and L/100 rats (Fig. 3a, lower panels). The integrated TLR-4/EGFR signaling culminates in the activation of ERK1/2 and NF-κB [37, 38], and accordingly, a 2-fold increase in the number of AECs with phosphorylated-ERK1/2 (pERK) was detected in L/100 compared with S/100 rats (Fig. 3b) with no discernable change in the NF-κB p65 levels (personal observation). These data suggest that airway epithelial cells have a stronger TLR4/EGFR signal at 40 d after initial exposure.

### Rapid mucous cell metaplasia in LPS-pretreated Foxn1^nu^ mice lacking T lymphocytes

**Fig. 4:**
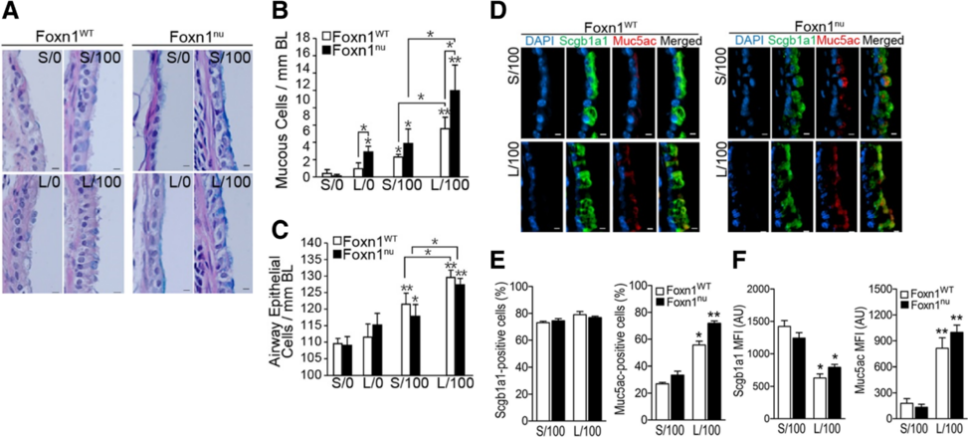
LPS-induced mucous cell metaplasia is augmented in LPS-pretreated athymic mice compared to euthymic mice. **a** Representative photomicrographs of axial airway sections stained with AB/H&E from S/0, S/100, L0 and L/100 Foxn1^WT^ mice compared with respective Foxn1^nu^ mice. Quantification of the number of mucous cells (**b**) and the number of airway epithelial cells (**c**) analyzed per mm BL. **d** Representative micrographs of axial airway sections showing Scgb1a1 (*green*) and Muc5ac (*red*) positivity with DAPI-stained nuclei (*blue*) in S/100 and L/100 Foxn1^WT^ and Foxn1^nu^ mice. **e** Quantification of the number of Scgb1a1- and Muc5ac-positive cells in S/100 and L/100 Foxn1^WT^ and Foxn1^nu^ mice. **f** Mean florescence intensity (MFI) of Scgb1a1- and Muc5ac-positive cells in S/100 and L/100 Foxn1^WT^ and Foxn1^nu^ mice. Error bar indicates mean ± SEM (*n* = 6 per group). * *P* < 0.05, ** *P* < 0.01 compared to S/0 unless otherwise indicated

In order to investigate the role of of T cells in epithelial memory to LPS we analyzed the T cell-deficient athymic nude (Foxn1^nu^) C57BL/6 mice in comparison with T cell-sufficient euthymic wild-type (Foxn1^WT^) C57BL/6 mice. The number of MCs/mm BL was 2-fold higher in L/0 Foxn1^nu^ than L/0 Foxn1^WT^ mice, and the LPS challenge caused a rapid increase in MCM independent of the genotype. However, MCs/mm BL were significantly higher in L/100 than S/100 mice of both genotypes (Fig. 4a and b). The total epithelial cell were higher in all mice following the LPS challenge but the L/100 Foxn1^nu^ and L/100 Foxn1^WT^ mice had significantly higher AECs compared to S/100 Foxn1^nu^ and S/100 Foxn1^WT^ mice (Fig. 4c).

Among the S/100 mice, Muc5ac expression was significantly increased and Scgb1a1 levels reduced in Foxn1^nu^ than in Foxn1^WT^ mice (Fig. 4d, e and f). Within the secretory cell populations, there was no difference in the number of Scgb1a1-positive club cells (ranging between 73 to 79 %) but the number of Muc5ac-positive cells were higher in Foxn1^nu^ (55.6 ± 2.8 %) than Foxn1^WT^ (71.9 ± 1.7 %) mice (Fig. 4e). However, LPS challenge increased the number of Muc5ac-positive cells especially in L/100 compared with S/100 mice in both genotypes (Fig. 4d and e). The expression levels of Scgb1a1 as measured by MFI was reduced 2-fold in the L/100 groups in both genotypes but Muc5ac MFI was increased greater than 4-fold (Fig. 4f).

### LPS pretreatment augments TLR4, EGFR, and nuclear pERK levels in mice lacking T cells

**Fig. 5:**
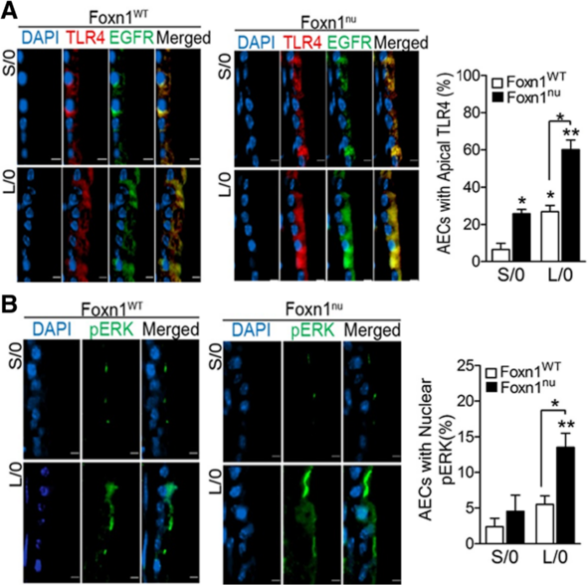
Expression of apical TLR4 and EGFR, and nuclear phosphorylated-ERK1/2 is augmented in LPS-pretreated athymic mice. **a** Quantification of the number of AECs with apical TLR4 and EGFR and representative micrographs of axial airways from S/0 and L/0 Foxn1^WT^ and Foxn1^nu^ mice. The panels are stained for DAPI (*blue*) nuclei, TLR4 (*red*), EGFR (*green*) and a merged image. **b** Quantification of the number of AECs with nuclear pERK in Foxn1^WT^ and Foxn1^nu^ mice. Representative micrographs of axial airways stained for pERK (*green*) and DAPI-stained (*blue*) nuclei from S/0 and L/0 Foxn1^WT^ and Foxn1^nu^ mice are shown. Error bar indicates mean ± SEM (*n* = 6 per group). * *P* < 0.05, ** *P* < 0.01 compared to Foxn1^WT^ S/0 unless otherwise indicated

Similar to rats, TLR4 and EGFR were expressed on basal and intraepithelial junctions of AECs in S/0 Foxn1^WT^ mice (Fig. 5a, upper panels). However, in S/0 Foxn1^nu^ mice expression was apical and increased (Fig. 5a, upper panels). LPS-pretreatment augmented the apical expression of TLR4 and EGFR in both L/0 mice but more prominently in Foxn1^nu^ than in Foxn1^WT^ mice (Fig. 5a, lower panels). The number of AECs with nuclear pERK was similar among S/0 Foxn1^WT^ and Foxn1^nu^ mice (Fig. 5b, lower panels). However, a 2-fold higher number of AECs with nuclear pERK was detected in L/0 Foxn1^nu^ mice than in L/0 Foxn1^WT^ mice (Fig. 5b, lower panels). This difference was no longer observed after the LPS challenge as all mice showed significantly higher number of AECs with nuclear pERK (Additional file 1: Figure S1).

## Discussion

The present study shows that airway epithelial cells that have been previously primed with LPS respond to subsequent LPS challenge with a rapid increase in MCM and epithelial cell hyperplasia (ECH). This memory response may be due to a sustained spatiotemporal localization of TLR4 and EGFR that initiate a rapid mucus expression via ERK1/2 activation.

Innate memory-based responses have been described in several immune cell populations including monocytes, macrophages and NK cells [39–41]. Recent seminal studies have highlighted the role of memory-based or ‘trained’ immune responses as summarized recently by Netea et al. [42]. These trained responses are adaptive and beneficial because mice primed with microbial ligands or PRRs are protected against subsequent lethal infection [39, 43, 44]. These trained responses have been mechanistically attributed to the transcriptional and epigenetic reprogramming that involved histone modifications but DNA methylation, microRNA and/or long noncoding RNA could also be implicated [39, 45]. Future studies will investigate the molecular basis of reprogramming in AECs that resulted in the observed augmented memory-based or trained responses.

Airway epithelial responses including MCM, ECH, and nuclear localization of pERK were elevated in L/0 rats compared with L/0 wild-type mice. This disparity could be due to differences in epithelial cell types present in the conducting airway epithelium. At airway generation 5, the site for all quantifications in the current study, serous cells are present in rats but absent in mice [7]. Further, mice in general respond with minimal mucous cell metaplasia in response to many insults possibly due to a more resistant genotype that results in less sustained activation of the signaling pathways and transcription factors compared to rats [31]. This was also evident by the dose of LPS used for challenge because 1 μg LPS caused MCM in LPS-pretreated rats (L/1) but had no discernible airway epithelial changes in mice irrespective of the LPS priming (personal observation). As was previously reported [8, 9], Muc5ac-positive mucous cells differentiate from the Scgb1a1-positive club cells. Consistent with these observations, a marked suppression of Scgb1a1 expression was observed by us following the LPS challenge in all rodents.

T cells affect the generation of mucous cell metaplasia in response to various challenges [23, 46]. Based on the threshold and type of insult, the immunologic tolerance dominates the primary response and is generally driven in part by T cells, specifically by resident memory (T_m_) and regulatory (T_reg_) T cells [23, 47, 48]. Therefore, to isolate the epithelial memory responses from that of T cells we analyzed the secondary response in the absence of T cells. Moreover the memory-based immune responses have also been previously demonstrated as lymphocyte-independent mechanism using athymic and *Rag1*-deficient mice [39, 49]. We found that athymic Foxn1^nu^ mice responded with enhanced MCM in response to LPS or saline challenge compared to Foxn1^WT^ mice. Higher apical expression of TLR4 and EGFR in Foxn1^nu^ mice that rapidly activates ERK1/2 phosphorylation and nuclear localization may be involved in this process. Validation of these findings by in situ hybridization or q-PCR from microdissected airway cells would require more comprehensive kinetic (several time-point) analyses to adequately match the observed changes at protein levels.

The L/0 Foxn1^nu^ mice showed higher number of mucous cells even when instilled with saline as a secondary challenge (Fig. 4b) with a corresponding increased TLR4 expression compared to L/0 Foxn1^WT^ mice (Fig. 5a). We believe that these mice are more responsive to saline challenge and, therefore, showed increased mucous cell numbers and TLR4 expression. Form our previous studies, we have not observed differences in mucous cells at baseline, and we assume that 40 d after LPS challenge all mucous cells were resolved also in Foxn1^nu^ mice as in wild-type mice. Other studies have also documented that following a primary LPS exposure there were no discernible differences reported in athymic mice lacking T lymphocytes compared to euthymic wild-type mice [50]. Our previous studies were focused on the kinetics of LPS response in rats, and the T cells along with other immune cells returned back to normal levels by day 40 [17].

T_reg_ or T_m_ cells may suppress TLR4 and EGFR expression in AECs to establish a tolerogenic homeostasis in airway mucosa [23, 47, 48]. Future studies will investigate which T cell sub-population contributes to the suppression of apical TLR4 and EGFR expression on AECs. In vitro studies using air-liquid interface cultures of AECs will also help determine if this memory-response in AECs requires a signal from T cells to suppress LPS-induced TLR4 expression.

The number of AECs was increased in LPS-pretreated rats one day after saline challenge. Because all inflammation was resolved by day 40 with epithelial cell numbers returning the levels found in naïve rats by day 30 [17], this rapid increase in ECH by saline instillation suggests that LPS-primed airway epithelial cells can proliferate rapidly. Whether the observed ‘innate memory’ that was established in response to LPS instillation may also respond to a secondary challenge by insults other than LPS will be investigated in the future. TLR4 is one of the pattern-recognition receptors (PRRs) that regulate maladaptive immune response to LPS [51]. LPS responsiveness is fine-tuned by the levels of TLR4 present on the cell surface which in turn is determined by the amount of TLR4 trafficking between the Golgi and the plasma membrane, and the TLR4 internalized into endosomes [52]. Dysregulation of TLR4 expression or localization at the epithelial interface results in impaired host response to LPS as observed in cystic fibrosis [51]. TLR4 integrates its signaling with EGFR and helps regulate the proliferative responses [53–55]. Interestingly, in athymic Foxn1^nu^ mice, the levels of TLR4 and EGFR were augmented with some AECs showing nuclear pERK in LPS-pretreated mice even in the absence of LPS challenge. Therefore, EGFR and ERK1/2, the known inducers of MCM [35–38] could be responsible for the ‘memory’ in airway epithelial cells.

Mucous cell metaplasia is a reversible adaptive response that increases mucous secretions to help clear the airways through mucociliary clearance. However, whether these metaplastic cells retain a memory to a prior exposure has not been previously investigated. We and others have reported that the hyperplastic epithelial cells undergo apoptosis during the resolution process [17, 56–58]. Whether some of the hyperplastic cells remain following the resolution process and serve as ‘memory’ cells remains to be investigated. In addition, AECs secrete mucous and inflammatory factors, and interact with other epithelial and mucosal immune cells in an autocrine and a paracrine manner. The mucus layer helps trap inhaled toxicants and dilutes local inflammatory factors or chemoattractants to suppress the effect on other epithelial and immune cells. Thus, the intricate and tightly regulated crosstalk between immune and epithelial cells may be required to adjust a tolerant versus a hyperreactive epithelium. The reported suppressive role of T cells on the hyperactive mucous response could also help understand the pulmonary responses to inhaled toxicants that result in chronic mucous hypersecretion in certain susceptible and immunocompromised population.

## Conclusions

The findings suggest that airway epithelial cells preserve an ‘innate memory’ to previous LPS exposure via a sustained expression of TLR4 and EGFR that help potentiate a rapid mucous response following a secondary challenge. This memory-based or ‘trained’ response of AECs was suppressed by T lymphocytes because there was further augmented mucous response in athymic mice. This airway epithelial and T cells nexus may play a role in aberrant mucous cell differentiation and mucin hypersecretion observed in chronic airway diseases.

## Additional file

Additional file 1:
**Figure S1.** Quantification of AECs with nuclear pERK in Foxn1^WT^ and Foxn1^nu^ mice following LPS challenge. Representative micrographs of axial airways stained for pERK (green) and DAPI-stained (blue) nuclei from S/100 and L/100 Foxn1^WT^ and Foxn1^nu^ mice. Error bar indicates mean ± SEM (*n* = 6 per group). * *P* < 0.05. (DOCX 1546 kb)

## Abbreviations

AECs: Airway epithelial cells
BL: Basal lamina
ECH: Epithelial cell hyperplasia
EGFR: Epidermal growth factor receptor
ERK: Extracellular-signal regulated kinase
LPS: Lipopolysaccharide
MAPK: Mitogen activate protein kinase
MC: Mucous cells
MCM: Mucous cell metaplasia
MFI: Mean fluorescence intensity
pERK: phosphorylated extracellular-signal regulated kinase
TLR4: Toll-like receptor 4
V_s_: Volume of stored mucosubstances

## References

[1] Whitsett JA, Alenghat T (2015). Respiratory epithelial cells orchestrate pulmonary innate immunity. Nat Immunol.

[2] Hammad H, Lambrecht BN (2015). Barrier Epithelial Cells and the Control of Type 2 Immunity. Immunity.

[3] Weitnauer M, Mijosek V, Dalpke AH (2016). Control of local immunity by airway epithelial cells. Mucosal Immunol.

[4] McCauley HA, Guasch G (2015). Three cheers for the goblet cell: maintaining homeostasis in mucosal epithelia. Trends Mol Med.

[5] Adler KB, Tuvim MJ, Dickey BF (2013). Regulated mucin secretion from airway epithelial cells. Front Endocrinol (Lausanne).

[6] Hogan BL, Barkauskas CE, Chapman HA, Epstein JA, Jain R, Hsia CC, Niklason L, Calle E, Le A, Randell SH (2014). Repair and regeneration of the respiratory system: complexity, plasticity, and mechanisms of lung stem cell function. Cell Stem Cell.

[7] Tesfaigzi Y, Harris JF, Hotchkiss JA, Harkema JR (2004). DNA synthesis and Bcl-2 expression during development of mucous cell metaplasia in airway epithelium of rats exposed to LPS. Am J Physiol Lung Cell Mol Physiol.

[8] Evans CM, Williams OW, Tuvim MJ, Nigam R, Mixides GP, Blackburn MR, DeMayo FJ, Burns AR, Smith C, Reynolds SD (2004). Mucin is produced by clara cells in the proximal airways of antigen-challenged mice. Am J Respir Cell Mol Biol.

[9] Reader JR, Tepper JS, Schelegle ES, Aldrich MC, Putney LF, Pfeiffer JW, Hyde DM (2003). Pathogenesis of mucous cell metaplasia in a murine asthma model. Am J Pathol.

[10] Tata PR, Mou H, Pardo-Saganta A, Zhao R, Prabhu M, Law BM, Vinarsky V, Cho JL, Breton S, Sahay A (2013). Dedifferentiation of committed epithelial cells into stem cells in vivo. Nature.

[11] Fahy JV, Dickey BF (2010). Airway mucus function and dysfunction. N Engl J Med.

[12] Rose MC, Voynow JA (2006). Respiratory tract mucin genes and mucin glycoproteins in health and disease. Physiol Rev.

[13] Thai P, Loukoianov A, Wachi S, Wu R (2008). Regulation of airway mucin gene expression. Annu Rev Physiol.

[14] Lillehoj EP, Kato K, Lu W, Kim KC (2013). Cellular and molecular biology of airway mucins. Int Rev Cell Mol Biol.

[15] Evans CM, Raclawska DS, Ttofali F, Liptzin DR, Fletcher AA, Harper DN, McGing MA, McElwee MM, Williams OW, Sanchez E (2015). The polymeric mucin Muc5ac is required for allergic airway hyperreactivity. Nat Commun.

[16] Young HW, Williams OW, Chandra D, Bellinghausen LK, Perez G, Suarez A, Tuvim MJ, Roy MG, Alexander SN, Moghaddam SJ (2007). Central role of Muc5ac expression in mucous metaplasia and its regulation by conserved 5’ elements. Am J Respir Cell Mol Biol.

[17] Harris JF, Aden J, Lyons CR, Tesfaigzi Y (2007). Resolution of LPS-induced airway inflammation and goblet cell hyperplasia is independent of IL-18. Respir Res.

[18] Li L, Jacinto R, Yoza B, McCall CE (2003). Distinct post-receptor alterations generate gene- and signal-selective adaptation and cross-adaptation of TLR4 and TLR2 in human leukocytes. J Endotoxin Res.

[19] Glass CK, Saijo K (2010). Nuclear receptor transrepression pathways that regulate inflammation in macrophages and T cells. Nat Rev Immunol.

[20] Lamagna C, Scapini P, van Ziffle JA, DeFranco AL, Lowell CA (2013). Hyperactivated MyD88 signaling in dendritic cells, through specific deletion of Lyn kinase, causes severe autoimmunity and inflammation. Proc Natl Acad Sci U S A.

[21] Morris MC, Gilliam EA, Li L (2014). Innate immune programing by endotoxin and its pathological consequences. Front Immunol.

[22] Curtis JL (2005). Cell-mediated adaptive immune defense of the lungs. Proc Am Thorac Soc.

[23] Vernooy JH, Dentener MA, van Suylen RJ, Buurman WA, Wouters EF (2002). Long-term intratracheal lipopolysaccharide exposure in mice results in chronic lung inflammation and persistent pathology. Am J Respir Cell Mol Biol.

[24] Dong L, Li H, Wang S, Li Y (2009). Different doses of lipopolysaccharides regulate the lung inflammation of asthmatic mice via TLR4 pathway in alveolar macrophages. J Asthma.

[25] Willart MA, Lambrecht BN (2009). The danger within: endogenous danger signals, atopy and asthma. Clin Exp Allergy.

[26] Silva MA, Bercik P (2012). Macrophages are related to goblet cell hyperplasia and induce MUC5B but not MUC5AC in human bronchus epithelial cells. Lab Invest.

[27] Harkema JR, Hotchkiss JA, Barr EB, Bennett CB, Gallup M, Lee JK, Basbaum C (1999). Long-lasting effects of chronic ozone exposure on rat nasal epithelium. Am J Respir Cell Mol Biol.

[28] Harada K, Isse K, Sato Y, Ozaki S, Nakanuma Y (2006). Endotoxin tolerance in human intrahepatic biliary epithelial cells is induced by upregulation of IRAK-M. Liver Int.

[29] Chand HS, Harris JF, Mebratu Y, Chen Y, Wright PS, Randell SH, Tesfaigzi Y (2012). Intracellular insulin-like growth factor-1 induces Bcl-2 expression in airway epithelial cells. J Immunol.

[30] Chand HS, Woldegiorgis Z, Schwalm K, McDonald J, Tesfaigzi Y (2012). Acute Inflammation Induces IGF-1 to Mediate Bcl-2 and Muc5ac Expression in Airway Epithelial Cells. Am J Respir Cell Mol Biol.

[31] Chand HS, Montano G, Huang X, Randell SH, Mebratu Y, Petersen H, Tesfaigzi Y (2014). A genetic variant of p53 restricts the mucous secretory phenotype by regulating SPDEF and Bcl-2 expression. Nat Commun.

[32] McAlees JW, Whitehead GS, Harley IT, Cappelletti M, Rewerts CL, Holdcroft AM, Divanovic S, Wills-Karp M, Finkelman FD, Karp CL, Cook DN (2015). Distinct Tlr4-expressing cell compartments control neutrophilic and eosinophilic airway inflammation. Mucosal Immunol.

[33] Lu YC, Yeh WC, Ohashi PS (2008). LPS/TLR4 signal transduction pathway. Cytokine.

[34] Schwartz DA (2001). The role of TLR4 in endotoxin responsiveness in humans. J Endotoxin Res.

[35] Tyner JW, Kim EY, Ide K, Pelletier MR, Roswit WT, Morton JD, Battaile JT, Patel AC, Patterson GA, Castro M (2006). Blocking airway mucous cell metaplasia by inhibiting EGFR antiapoptosis and IL-13 transdifferentiation signals. J Clin Invest.

[36] Burgel PR, Nadel JA (2008). Epidermal growth factor receptor-mediated innate immune responses and their roles in airway diseases. Eur Respir J.

[37] Ha U, Lim JH, Jono H, Koga T, Srivastava A, Malley R, Pages G, Pouyssegur J, Li JD (2007). A novel role for IkappaB kinase (IKK) alpha and IKKbeta in ERK-dependent up-regulation of MUC5AC mucin transcription by Streptococcus pneumoniae. J Immunol.

[38] Ohnishi H, Takeda K, Domenico J, Lucas JJ, Miyahara N, Swasey CH, Dakhama A, Gelfand EW (2009). Mitogen-activated protein kinase/extracellular signal-regulated kinase 1/2-dependent pathways are essential for CD8+ T cell-mediated airway hyperresponsiveness and inflammation. J Allergy Clin Immunol.

[39] Quintin J, Saeed S, Martens JH, Giamarellos-Bourboulis EJ, Ifrim DC, Logie C, Jacobs L, Jansen T, Kullberg BJ, Wijmenga C (2012). Candida albicans infection affords protection against reinfection via functional reprogramming of monocytes. Cell Host Microbe..

[40] Sun JC, Madera S, Bezman NA, Beilke JN, Kaplan MH, Lanier LL (2012). Proinflammatory cytokine signaling required for the generation of natural killer cell memory. J Exp Med..

[41] Chen F, Wu W, Millman A, Craft JF, Chen E, Patel N, Boucher JL, Urban JF, Kim CC, Gause WC (2014). Neutrophils prime a long-lived effector macrophage phenotype that mediates accelerated helminth expulsion. Nat Immunol..

[42] Netea MG, Joosten LA, Latz E, Mills KH, Natoli G, Stunnenberg HG, O’Neill LA, Xavier RJ. Trained immunity: a program of innate immune memory in health and disease. Science. 2016;352:aaf1098. 10.1126/science.aaf1098 PMC5087274 27102489

[43] Munoz N, Van Maele L, Marques JM, Rial A, Sirard JC, Chabalgoity JA (2010). Mucosal administration of flagellin protects mice from Streptococcus pneumoniae lung infection. Infect Immun..

[44] Zhang B, Chassaing B, Shi Z, Uchiyama R, Zhang Z, Denning TL, Crawford SE, Pruijssers AJ, Iskarpatyoti JA, Estes MK (2014). Viral infection. Prevention and cure of rotavirus infection via TLR5/NLRC4-mediated production of IL-22 and IL-18. Science..

[45] Saeed S, Quintin J, Kerstens HH, Rao NA, Aghajanirefah A, Matarese F, Cheng SC, Ratter J, Berentsen K, van der Ent MA (2014). Epigenetic programming of monocyte-to-macrophage differentiation and trained innate immunity. Science..

[46] Borchers MT, Wesselkamper SC, Deshmukh H, Beckman E, Medvedovic M, Sartor M, Leikauf GD, Committee HEIHR (2009). The role of T cells in the regulation of acrolein-induced pulmonary inflammation and epithelial-cell pathology. Res Rep Health Eff Inst..

[47] Lei L, Zhang Y, Yao W, Kaplan MH, Zhou B (2011). Thymic stromal lymphopoietin interferes with airway tolerance by suppressing the generation of antigen-specific regulatory T cells. J Immunol.

[48] DiCosmo BF, Geba GP, Picarella D, Elias JA, Rankin JA, Stripp BR, Whitsett JA, Flavell RA (1994). Airway epithelial cell expression of interleukin-6 in transgenic mice. Uncoupling of airway inflammation and bronchial hyperreactivity. J Clin Invest.

[49] Bistoni F, Verducci G, Perito S, Vecchiarelli A, Puccetti P, Marconi P, Cassone A (1988). Immunomodulation by a low-virulence, agerminative variant of Candida albicans. Further evidence for macrophage activation as one of the effector mechanisms of nonspecific anti-infectious protection. J Med Vet Mycol.

[50] Morris PE, Glass J, Cross R, Cohen DA (1997). Role of T-lymphocytes in the resolution of endotoxin-induced lung injury. Inflammation.

[51] John G, Yildirim AO, Rubin BK, Gruenert DC, Henke MO (2010). TLR-4-mediated innate immunity is reduced in cystic fibrosis airway cells. Am J Respir Cell Mol Biol.

[52] McGettrick AF, O’Neill LA (2010). Localisation and trafficking of Toll-like receptors: an important mode of regulation. Curr Opin Immunol.

[53] De S, Zhou H, DeSantis D, Croniger CM, Li X, Stark GR (2015). Erlotinib protects against LPS-induced endotoxicity because TLR4 needs EGFR to signal. Proc Natl Acad Sci U S A.

[54] Chattopadhyay S, Veleeparambil M, Poddar D, Abdulkhalek S, Bandyopadhyay SK, Fensterl V, Sen GC (2015). EGFR kinase activity is required for TLR4 signaling and the septic shock response. EMBO Rep.

[55] Koff JL, Shao MX, Ueki IF, Nadel JA (2008). Multiple TLRs activate EGFR via a signaling cascade to produce innate immune responses in airway epithelium. Am J Physiol Lung Cell Mol Physiol.

[56] Mebratu YA, Dickey BF, Evans C, Tesfaigzi Y (2008). The BH3-only protein Bik/Blk/Nbk inhibits nuclear translocation of activated ERK1/2 to mediate IFNgamma-induced cell death. J Cell Biol.

[57] Mebratu YA, Schwalm K, Smith KR, Schuyler M, Tesfaigzi Y (2011). Cigarette smoke suppresses Bik to cause epithelial cell hyperplasia and mucous cell metaplasia. Am J Respir Crit Care Med.

[58] Nyunoya T, Mebratu Y, Contreras A, Delgado M, Chand HS, Tesfaigzi Y (2014). Molecular processes that drive cigarette smoke-induced epithelial cell fate of the lung. Am J Respir Cell Mol Biol.

