# Supplementary material for: T cells suppress memory-dependent rapid mucous cell metaplasia in mouse airways
Source: Respir Res. 2016 Oct 20;17:132. doi: 10.1186/s12931-016-0446-0 (PMC5073838; doi:10.1186/s12931-016-0446-0)
Supplement: Additional file 1: — Figure S1. Quantification of AECs with nuclear pERK in Foxn1WT and Foxn1nu mice following LPS challenge. Representative micrographs of axial airways stained for pERK (green) and DAPI-stained (blue) nuclei from S/100 and L/100 Foxn1WT and Foxn1nu mice. Error bar indicates mean ± SEM (n = 6 per group). * P < 0.05. (DOCX 1546 kb) [file 12931_2016_446_MOESM1_ESM.docx]

**Additional file 1**

T Cells Suppress Memory-Dependent Rapid Mucous Cell Metaplasia in Mouse Airways

Hitendra S. Chand†, Yohannes A. Mebratu, Marena Montera, and Yohannes Tesfaigzi

COPD Program, Lovelace Respiratory Research Institute, Albuquerque, NM - 87108, USA

† Present Address

Department of Immunology,

Florida International University

11200 SW 8th St,

Miami, FL 33199

Tel: (305) 348-1472

Fax: (305) 348-1109

E-mail: [hchand@fiu.edu](mailto:hchand@fiu.edu)

**
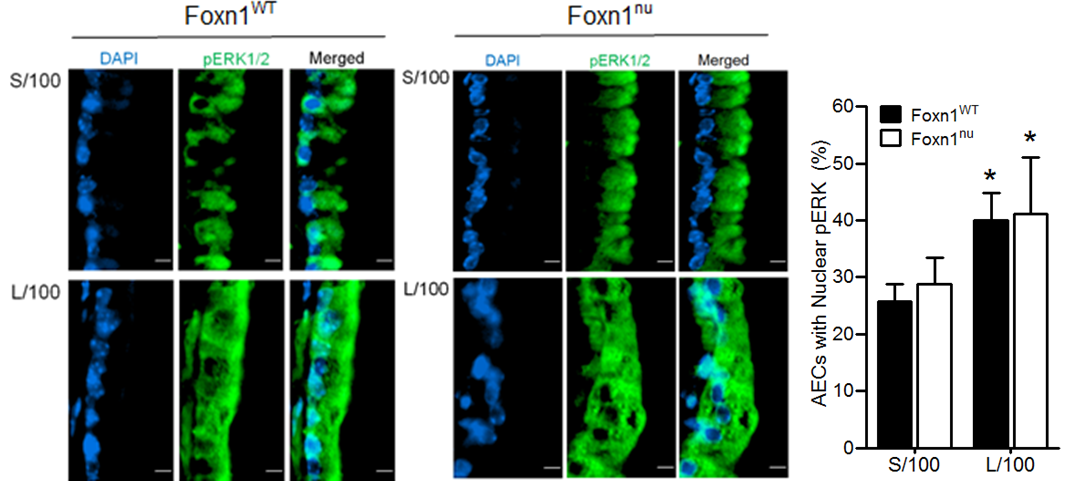
**

**Figure S1.** Quantification of AECs with nuclear pERK in Foxn1^WT^ and Foxn1^nu^ mice following LPS challenge. Representative micrographs of axial airways stained for pERK (green) and DAPI-stained (blue) nuclei from S/100 and L/100 Foxn1^WT^ and Foxn1^nu^ mice. Error bar indicates mean± SEM (n=6 per group). * *P* < 0.05.
